# Rise of multiple insecticide resistance in *Anopheles funestus* in Malawi: a major concern for malaria vector control

**DOI:** 10.1186/s12936-015-0877-y

**Published:** 2015-09-15

**Authors:** Jacob M. Riveron, Martin Chiumia, Benjamin D. Menze, Kayla G. Barnes, Helen Irving, Sulaiman S. Ibrahim, Gareth D. Weedall, Themba Mzilahowa, Charles S. Wondji

**Affiliations:** Vector Biology Department, Liverpool School of Tropical Medicine, Pembroke Place, Liverpool, UK; Malaria Alert Centre, College of Medicine, University of Malawi, Blantyre, Malawi; Organisation de Coordination pour la lutte contre les Endémies en Afrique Centrale, PO Box 288, Yaoundé, Cameroon

**Keywords:** Malaria, Insecticide resistance, Vector control, *Anopheles funestus*, Malawi

## Abstract

**Background:**

Deciphering the dynamics and evolution of insecticide resistance in malaria vectors is crucial for successful vector control. This study reports an increase of resistance intensity and a rise of multiple insecticide resistance in *Anopheles funestus* in Malawi leading to reduced bed net efficacy.

**Methods:**

*Anopheles funestus* group mosquitoes were collected in southern Malawi and the species composition, *Plasmodium* infection rate, susceptibility to insecticides and molecular bases of the resistance were analysed.

**Results:**

Mosquito collection revealed a predominance of *An. funestus* group mosquitoes with a high hybrid rate (12.2 %) suggesting extensive species hybridization. *An. funestus* sensu stricto was the main *Plasmodium* vector (4.8 % infection). Consistently high levels of resistance to pyrethroid and carbamate insecticides were recorded and had increased between 2009 and 2014. Furthermore, the 2014 collection exhibited multiple insecticide resistance, notably to DDT, contrary to 2009. Increased pyrethroid resistance correlates with reduced efficacy of bed nets (<5 % mortality by Olyset^®^ net), which can compromise control efforts. This change in resistance dynamics is mirrored by prevalent resistance mechanisms, firstly with increased over-expression of key pyrethroid resistance genes (*CYP6Pa*/*b* and *CYP6M7*) in 2014 and secondly, detection of the A296S-RDL dieldrin resistance mutation for the first time. However, the L119F-GSTe2 and *kdr* mutations were absent.

**Conclusions:**

Such increased resistance levels and rise of multiple resistance highlight the need to rapidly implement resistance management strategies to preserve the effectiveness of existing insecticide-based control interventions.

**Electronic supplementary material:**

The online version of this article (doi:10.1186/s12936-015-0877-y) contains supplementary material, which is available to authorized users.

## Background

Malaria remains a major public health burden in Africa [1], notably in Malawi, where it is highly endemic with an estimated six million annual cases [2, 3]. Current malaria control efforts in Malawi rely heavily on insecticide-based interventions such as long-lasting insecticide-treated nets (LLINs) and indoor residual spraying (IRS) [4]. However, reports of increasing resistance against the main insecticides used in public health are of concern for the continued effectiveness of these control tools. In Malawi, the concern is greater for the increasing cases of resistance against pyrethroids (the only insecticide class used in bed nets) reported in the major malaria vector *Anopheles funestus* [5–8]. Insecticide resistance is a dynamic process and resistance pattern can change rapidly with time, as reported in other vector species such as *Anopheles gambiae* [9, 10]. However, changes due to ongoing control programmes in the profiles, intensity and underlying resistance mechanisms in Malawi remain largely uncharacterized. Understanding the dynamics and evolution of the resistance pattern in such a major malaria vector is crucial for the design and implementation of successful resistance management strategies.

Design of effective control strategies relies also on a good knowledge of the vector population in term of species composition, vectorial capacity and behaviour. Such information remains patchy in Malawi, notably in the southern region where resistance has previously been reported [6]. *An. funestus* belongs to a group of ten to 11 species morphologically indistinguishable as adults [11]. However, the local species composition of this group, their role in malaria transmission, the hybridization between these species, and its impact on the introgression of genes of interests, such as resistance genes, remains largely uncharacterized.

To fill this knowledge gap and to facilitate the design and implementation of suitable vector control strategies, this study reports an extensive investigation of the dynamic changes in resistance profile and resistance mechanisms associated with ongoing insecticide-based control interventions in Malawi between 2009 and 2014. This study reveals an increase of resistance intensity and a rise of multiple insecticide resistance in *An. funestus* in Malawi causing a reduction in bed net efficacy.

## Methods

### Study area and mosquito collection

Adult *Anopheles* and *Culex* mosquitoes were collected in Chikwawa district (16°1′S; 34°47′E), southern Malawi, in January 2014. Geographical details of this location have been described previously [7]. Indoor-resting, blood-fed or gravid mosquitoes were collected between 06.00 and 12.00 h inside households using electric insect aspirators, after obtaining the consent of village chiefs and house owners. Collected mosquitoes were kept until fully gravid and induced to lay eggs in individual 1.5-ml microcentrifuge tubes, as described previously [12]. All F_0_ females that laid eggs were morphologically identified as belonging to either the *An. funestus* group or the *An. gambiae* complex according to a morphological key [13]. Dead adult mosquitoes and egg batches were transported to the Liverpool School of Tropical Medicine under a DEFRA license (PATH/125/2012).

### Species identification

To identify the different species within the *An. funestus* group, a cocktail PCR was performed as previously described [14] after genomic DNA extraction from whole mosquitoes using the DNeasy Blood and Tissue kit (Qiagen. Hilden, Germany). In addition, 50 females belonging to the *An. gambiae* complex were identified as previously described [15] after gDNA extraction [16]. Eggs were hatched in small paper cups and larvae transferred to plastic larvae trays, according to species, for rearing as previously described [12, 17].

### *Plasmodium* sporozoite infection rate

The *Plasmodium* infection rate of *An. funestus* group mosquitoes (167 *An. funestus*, s.s., 91 *Anopheles rivulorum*-like and 37 *An. rivulorum* females) was determined using a TaqMan assay to detect four *Plasmodium* species: *Plasmodiumfalciparum*, *Plasmodiumvivax*, *Plasmodiumovale*, and *Plasmodiummalariae* [18, 19].

### Insecticide susceptibility assays

Insecticide susceptibility bioassays were performed following WHO protocols [20] at 25 ± 2 °C and 70–80 % relative humidity. Assays were carried out with at least four replicates, with 25 individuals per tube, except for *An. gambiae* s.l. where only one assay per insecticide was carried out due to limited sample size. Susceptibility to ten insecticides belonging to the four major public health insecticide classes was tested in *An. funestus*: the pyrethroids permethrin (0.75 %), deltamethrin (0.05 %), lambda-cyhalothrin (0.05 %), and etofenprox (0.05 %); the carbamates bendiocarb (0.1 %) and propoxur (0.1 %); the organochlorines DDT (4 %) and dieldrin (4 %); and, the organophosphates malathion (5 %) and fenithrothion (1 %). Insecticide-impregnated papers were supplied by the WHO with the exception of etofenprox- and propoxur-impregnated papers, which were custom prepared following the standard WHO method. Two to five-day old F_1_ male and female adults were exposed to insecticide-treated papers for 60 min and then held in bioassay tubes with access to 10 % sugar solution. The mortality rate was determined 24 h after exposure. In each bioassay, a control experiment (using papers impregnated only with insecticide carrier oil) was performed following the same procedure.

#### Piperonyl butoxide (PBO) synergist assays

*Anopheles funestus* s.s. F_1_ adults were pre-exposed to PBO (4 %) impregnated paper for 1 h and thereafter immediately exposed to DDT (4 %) for another hour. Mortality was scored after 24 h and compared to the results obtained using DDT without PBO.

#### Resistance intensity

The LT_50_ (time required for 50 % mortality) of the Chikwawa *An. funestus* s.s. population to permethrin (0.75 %), an insecticide commonly used in impregnated bed nets, was estimated after exposure of adult females at different independent timepoints: 30, 60, 90, 120, and 180 min (25 females per tube; 1–5 replicates per timepoint because of limited sample size). Because no insecticide-susceptible *An. funestus* s.s. strain was available, the resistance intensity was estimated by comparing the results with those for the *An. gambiae*-susceptible strain Kisumu (LT_50_ 7.7 min) [21].

### Bed net efficacy estimate using cone assays

In order to check the efficacy of conventional bed nets against the Chikwawa *An. funestus* population, a 3-min exposure bioassay was performed following WHO guidelines with minor modifications [22]. Five replicates of ten F_1_ females (2–5 days old) were placed in plastic cones attached to two commercial nets: the Olyset^®^ Net (containing 2 % permethrin) and the Olyset^®^ Plus net (containing 2 % permethrin combined with 1 % of the synergist PBO). Three replicates of ten F_1_ mosquitoes (2–5 days old) were exposed to an untreated net as a control. After 3 min exposure, the mosquitoes were placed in paper cups with cotton soaked in 10 % sugar solution. Knockdown was scored 1 h after exposure and mortality 24 h after exposure.

### Gene expression profile of major *Anopheles funestus* insecticide resistance genes

Previous efforts to characterize the mechanisms of insecticide resistance in Malawian *An. funestus* s.s. populations revealed that pyrethroid resistance is mainly driven by key cytochrome P450s genes such as *CYP6P9a*, *CYP6P9b* and *CYP6M7* [7, 23]. The expression profiles of these three genes and one glutathione-S transferase gene (*GSTe2*) associated with *An. funestus* DDT resistance in West Africa [24], were assessed using quantitative reverse transcription PCR (qRT-PCR). These expression profiles were compared to those obtained from the 2009 sample [6]. Total RNA was extracted from three batches of ten F_1_ permethrin or DDT-resistant females collected in 2014. RNA extraction, cDNA synthesis, qRT-PCR reactions and analysis were conducted as previously reported [7, 25]. The relative expression and fold-change of each target gene in 2014 and 2009 relative to the susceptible strain FANG was calculated according to the 2−ΔΔCT method, incorporating PCR efficiency [26] after normalization with the housekeeping genes RSP7 (ribosomal protein S7; VectorBase ID: AFUN007153-RA) and actin (VectorBase ID: AFUN006819).

### Investigation of the role of knockdown resistance mutations in pyrethroid and DDT resistance

A fragment spanning a portion of intron 19 and the entire exon 20 of the voltage-gated sodium channel gene (VGSC), including the 1014 codon associated with resistance in *An. gambiae*, was amplified and sequenced in ten permethrin-resistant and ten susceptible mosquitoes from Chikwawa and the same for DDT. PCR, sequencing and analysis were carried out as previously described [17, 27]. All DNA sequences were submitted to GenBank (Accession Number: KR337655:337726).

A TaqMan assay was performed to genotype the L1014F *kdr* mutation in *An. gambiae* s.l. samples according to Bass et al. [28]. Additionally, a fragment of the VGSC gene spanning exon 20 was sequenced in ten field-collected *An. gambiae* s.l. for further confirmation.

### Genotyping of L119F-GSTe2 and A296S-GABA receptor mutations

To assess the role of the L119F-GSTe2 mutation in DDT resistance in Chikwawa, a TaqMan assay was used to genotype 40 F_0_ field-collected mosquitoes and 20 DDT-susceptible and 20 DDT-resistant F_1_ mosquitoes, as described previously [24]. Likewise, the role in dieldrin resistance of the A296S-RDL mutation was assessed by a newly designed TaqMan assay used to genotype 40 F_0_ field-collected female mosquitoes and 20 dieldrin-susceptible and 16 dieldrin-resistant F_1_ mosquitoes. The primer and reporter sequences are shown in Additional file 1: Table S1.

## Results

### Mosquito species composition

More than 3000 blood-fed mosquitoes (90 % *An. funestus*, 5 % *An. gambiae* s.l. and 5 % *Culex* spp.) were collected in Chikwawa in January 2014. Of these, 512 gravid female adult *An. funestus*, 86 *An. gambiae* s.l. and 32 *Culex quinquefasciatus* were placed in 1.5-ml microcentrifuge tubes and forced to lay eggs (using a forced egg-laying method).

**Fig. 1:**
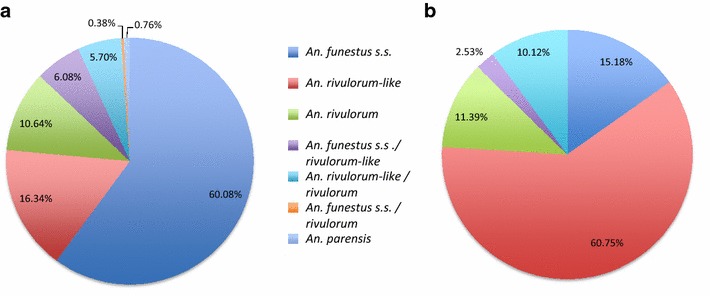
Species composition within the *Anopheles funestus* group in Chikwawa. **a** Females that laid eggs. **b** Females that did not lay eggs

From the 512 females of the *An. funestus* group, 263 females laid eggs and 249 females did not. Of the 263 females that laid eggs, *An. funestus* s.s. represented 60.1 % followed by *An. rivulorum*-like (16.3 %), *An. rivulorum* (10.6 %) and *Anopheles parensis* (0.76 %) (Fig. 1a; Additional file 2: Figure S1). Surprisingly, 32 individuals (12.2 %) were hybrids: 16 *An. funestus* s.s./*An. rivulorum*-like (6.1 %), one *An. funestus* s.s./*An. rivulorum* (0.4 %), and 15 *An. rivulorum*/*An. rivulorum*-like (5.7 %). Analysis of a batch of females that did not lay eggs (139 of the 249 females) detected the same species but at significantly different proportions. Among these, *An. rivulorum*-like was predominant (60.8 %), followed by *An. funestus* s.s. (15.2 %), *An. rivulorum* (11.4 %), the hybrids *An. rivulorum*/*An. rivulorum*-like (10.1 %), and *An. funestus* s.s./*An. rivulorum*-like (2.5 %) (Fig. 1b). In the case of *An. gambiae* complex mosquitoes, all 50 F_0_ females tested were identified as *Anopheles arabiensis*.

### *Plasmodium* infection rate

Only *P. falciparum* parasites were detected in the mosquitoes. They were detected in 4.8 % (8/167) of *An. funestus* s.s. and 1.07 % (1/93) of *An. rivulorum*-like.

### Insecticide susceptibility assays

Only *An. funestus* s.s. F_1_ progeny were successfully reared, as both *An. rivulorum* and *An. rivulorum*-like did not generate enough individuals for bioassay tests.

*Anopheles funestus* s.s. showed multiple resistance against different insecticide classes for both females and males. Resistance was particularly high against all pyrethroids, with very low mortality rates for permethrin (type I; ♀ 13 ± 5.3 % mortality) and deltamethrin (type II; ♀ 1.8 ± 1.8 %) and lambda-cyhalothrin (type II; ♀ 4.5 ± 0.3 %), whereas high resistance was observed against the non-ester pyrethroid etofenprox with no mortality.

**Fig. 2:**
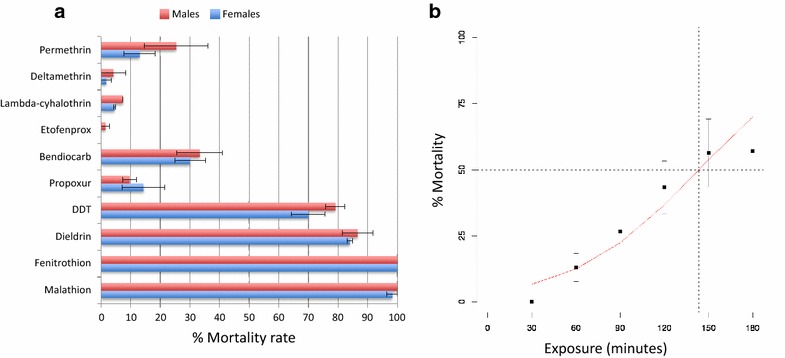
Resistance profile of the Chikwawa *An. funestus* s.s. population. **a** Mortality rates for different insecticides. **b** Timepoint mortality rates for permethrin. *Error bars* represent standard error of the mean

Similarly, this population is highly resistant to the carbamates bendiocarb (♀ 30.1 ± 5.1 % mortality) and propoxur (♀ 14.4 ± 7.2 %). Noticeably, the Chikwawa population has developed resistance to organochlorines. Unlike in 2009, it is now resistant to DDT (♀ 69.9 ± 5.7 % mortality) and to dieldrin (♀ 83.9 ± 0.9 %). However, it remains fully susceptible to organophosphates, with 100 % mortality for fenitrothion and 98.3 % for malathion (Fig. 2a).

### Resistance intensity

The LT_50_ of the Chikwawa population for permethrin was 143.5 min (females) (Fig. 2b) resulting in a resistance ratio of 18.6 (compared to susceptible *An. gambiae*).

### Change in resistance intensity between 2009 and 2014

**Fig. 3:**
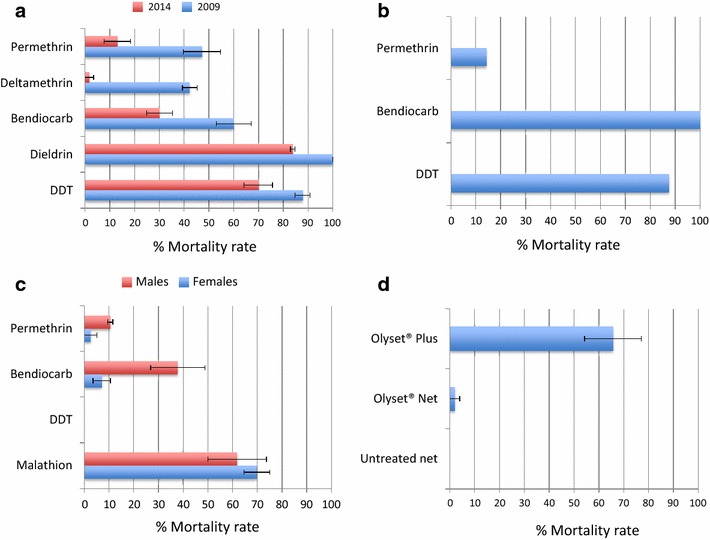
Dynamics and impact of resistance in Chikwawa. **a** Comparison of insecticide resistance profiles of *An. funestus* s.s. females collected in 2009 vs 2014; **b** resistance profile for the Chikwawa population of *An. arabiensis*; **c** *C. quinquefasciatus*; **d** efficacy assay results for Olyset^®^ and Olyset^®^ Plus bed nets. *Error bars* represent standard error of the mean

Comparison of resistance levels between 2009 and 2014 reveals that the resistance intensity has significantly increased in the *An. funestus* population of Chikwawa, notably for pyrethroids. For example, for females, the mortality on exposure to deltamethrin has decreased by 40.5 % (from 42.3 % in 2009 to just 1.8 % in 2014) and by 34.2 % for permethrin (from 47.2 % in 2009 to 13 % in 2014). Similar decreases of mortality rates are observed for the other insecticides, including the carbamate bendiocarb (29.9 % reduction from 60 % in 2009 to 30.1 % in 2014) and to a lesser extent the organochlorine DDT, with an 18.9 % reduction (87.8 % in 2009 to 69.9 % in 2014). Surprisingly, resistance to organochlorines also now extends to dieldrin, with a 16.1 % reduction in mortality from 100 % in 2009 to 83.9 % in 2014 (Fig. 3a).

### Insecticide susceptibility in *Anopheles arabiensis* and *Culex quinquefasciatus*

While the primary focus in this study is *An. funestus*, it is important to assess the impact of insecticide-based control interventions on other vector species, not least to compare differential effects of interventions among species. For this reason, this study also determined the insecticide resistance profiles of *An. arabiensis* and *C. quinquefasciatus*, two mosquito species that occur alongside *An. funestus* in Chikwawa, although in lower numbers*. Anopheles arabiensis* females were highly resistant to the pyrethroid permethrin (♀ 14.3 % mortality) and moderately resistant to the organochlorine DDT (♀ 87.5 % mortality), but fully susceptible to the carbamate bendiocarb (♀ 100 % mortality) (Fig. 3b). Significantly higher and multiple resistance to all four insecticide classes was observed in *C. quinquefasciatus* (Fig. 3c). For example, no mortality was observed for DDT.

### Synergist assay for DDT resistance

To test whether DDT resistance was mediated by the activity of cytochrome P450 genes, mosquitoes were exposed to DDT following exposure to the synergist PBO, an inhibitor of the activity of P450s. The PBO synergist assay revealed a high recovery of susceptibility to DDT after pre-exposure for 1 h to PBO (♀ 95.9 ± 3 %; ♂ 97.30 ± 1.2 % mortality) suggesting that cytochrome P450 genes might be playing a important role in this resistance.

### Insecticide-impregnated bed net efficacy assays

A nearly complete loss of efficacy was observed for the Olyset^®^ Net (2 % permethrin), with only 2 % mortality after 3 min exposure. A higher but not full efficacy was observed for the Olyset^®^ Plus (2 % permethrin plus 1 % PBO) net with 67 % mortality (Fig. 3d). These results support a key role for cytochrome P450s in the resistance to pyrethroids.

### Transcriptional profiling of metabolic resistance genes

**Fig. 4:**
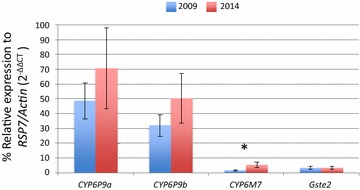
Differential expression of major insecticide resistance genes in the *An. funestus* s.s. Chikwawa population between 2009 and 2014, measured by qRT-PCR. *p < 0.05

Significant over-expression was observed in Chikwawa in 2014 for the cytochrome P450s *CYP6P9a* and *CYP6P9b*, known to confer pyrethroid resistance in *An. funestus* [7] compared to the susceptible strain FANG [fold change (FC) 70.6 and FC 50.3, respectively]. Comparison with 2009 levels revealed that the expression levels of these two genes have increased by a factor of 1.45 for *CYP6P9a* and 1.57 for *CYP6P9b* although the difference was not statistically significant. However, a significant increase in the expression of another pyrethroid resistance gene, the cytochrome P450 *CYP6M7* [23], was observed in the samples collected in 2014 compared to 2009 (FC 3.62 ± 1.206, P < 0.05) (Fig. 4). Nevertheless, expression levels of *CYP6M7* remain lower in Chikwawa compared to *CYP6P9a* and *CYP6P9b*, with only a FC of 5.28 ± 1.76 in 2014. No change was observed for the glutathione-S transferase, *GSTe2* as expression remains low (FC < 4) contrary to West Africa where it confers DDT and permethrin resistance [24].

### Role of knockdown resistance in pyrethroid and DDT resistance

**Fig. 5:**
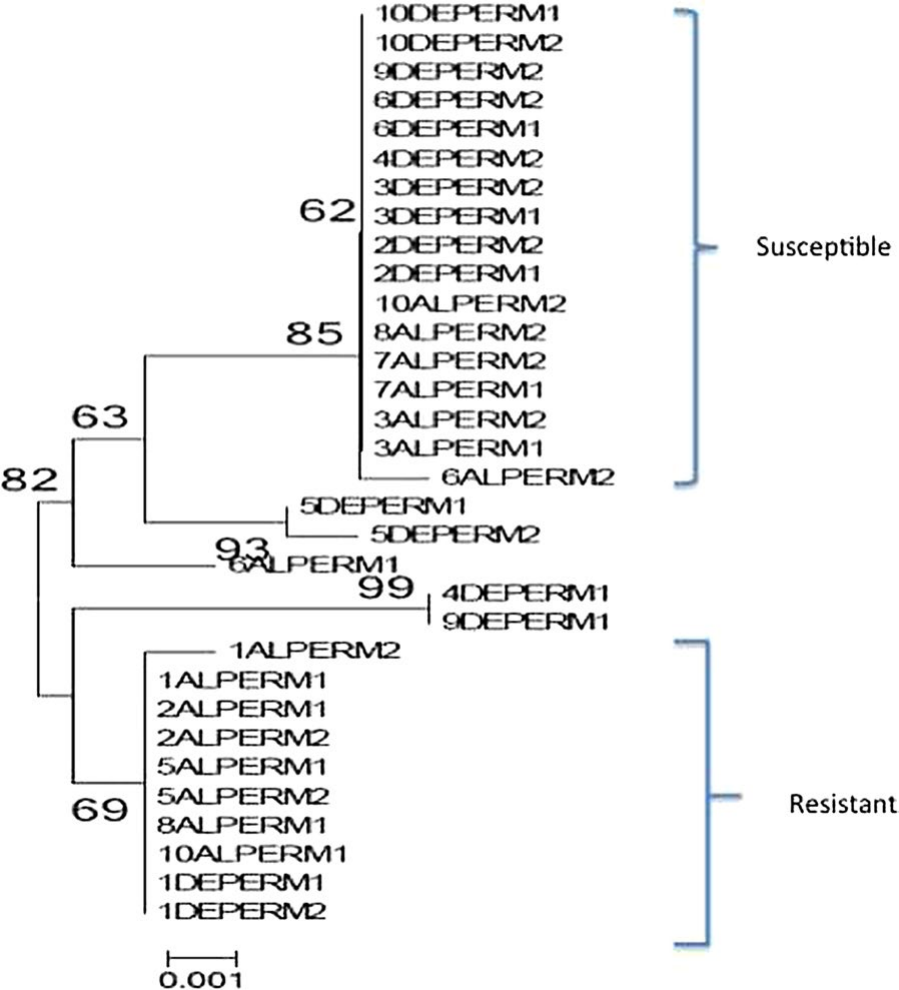
Maximum likelihood tree of VGSC haplotypes of permethrin-resistant and -susceptible *An. funestus* from Chikwawa. AL and DE denote mosquitoes alive or dead after insecticide exposure (i.e., resistant or susceptible). Perm denotes Permethrin

Neither the 1014 *kdr* mutation nor any mutation was detected from exon 20 of the VGSC gene (Additional file 3: Table S2). A clustering of haplotypes according to resistance phenotypes was observed for permethrin-exposed samples (Fig. 5) but not for the DDT-exposed samples (Additional file 4: Figure S2A, B), suggesting the presence of a novel *kdr* mutation in this population associated with permethrin resistance.

No 1014F mutation was detected in permethrin-resistant *An. arabiensis* by TaqMan or sequencing of the exon 20 of VGSC as previously reported in other populations of this species [29].

### Role of the L119F-GSTe2 in DDT resistance

All 40 F_0_ and 40 F_1_ (20 DDT resistant and 20 susceptible) mosquitoes genotyped were homozygous for the susceptible L119 allele (codon CTT) indicating that DDT resistance in Chikwawa is not conferred by the L119F-GSTe2 mutation.

### Detection of the A296S *RDL* mutation

Genotyping of 38 field-collected females detected the 296S *RDL*-resistant allele for the first time in a southern African *An. funestus* population at a frequency of 10.5 % and all occurring as heterozygotes. A significant association was observed between the 296S-resistant allele and dieldrin resistance as all susceptible mosquitoes were homozygous for the A296-susceptible allele whereas all resistant mosquitoes were heterozygous for 296S/A296 (odds ratio = infinity; P < 0.0001).

## Discussion

Assessing the impact of ongoing insecticide-based control interventions on natural populations of malaria vectors is important for the design of suitable resistance management strategies. The detection in this study of a significant increase of resistance levels and the rise of multiple insecticide resistance over a 5-year period in southern Malawi provides important information on the dynamics and evolution of insecticide resistance in a major malaria vector in an area with ongoing vector control.

### A complex vector population represents a challenge for malaria control

This study revealed that the composition of the *An. funestus* group in southern Malawi is more complex than previously reported, with four species identified and a large number of hybrids. A study in Karonga, in northern Malawi [8], found a similar diversity in the species composition, but did not detect *An. rivulorun*-like mosquitoes or hybrids [8]. The *An. funestus*-like, previously reported in Malawi [30], was not detected in this study despite the inclusion of primers to detect it in the species-typing PCR assay. Such diversity within the *An. funestus* group highlights the need for accurate species identification for this species group across Malawi to improve the reliability of entomological data generated from studies targeting *An. funestus* s.s., such as susceptibility levels to insecticides.

The high proportion of hybrids in this study suggests high levels of introgression between members of the *An. funestus* group. Such introgression could enable the exchange of genes of interest between these species, such as for susceptibility to *Plasmodium* infection or resistance to insecticides, possibly impacting upon their contribution to malaria transmission. This high hybridization rate between species of *An. funestus* group is similar to the high hybridization levels observed between *An. gambiae* s.s. and *Anopheles coluzzii* in the far west of their distribution range [31]. The underlying causes of such a high level of hybridization in Chikwawa should be further investigated.

### *Plasmodium* infection rate in southern Malawi

Of the species found, *An. funestus* s.s. is the most anthropophilic and endophilic mosquito [32], followed by *An. rivulorum*, which has previously been reported as a minor malaria vector in different African countries such as Tanzania [33] or Kenya [34]. In this study, the role of *An. funestus* s.s. in malaria transmission in southern Malawi is confirmed with infection rate similar to levels commonly reported for this species across Africa [35]. The role of *An. rivulorum* as a minor malaria vector could not be confirmed, but due to the low number of blood-fed females collected (only 37), its participation in malaria transmission cannot be ruled out either. Interestingly, this study shows that one *An. rivulorum*-like mosquito, considered mainly zoophilic and not involved in malaria transmission [36], was positive for *P. falciparum*. Further studies with higher numbers of samples are necessary to further assess the role of *An. rivulorum* and *An. rivulorum*-like mosquitoes in malaria transmission in southern Malawi.

### Development of multiple insecticide resistance in Malawian *Anopheles funestus*

This study revealed an increase in the level of resistance in a period of 5 years and also a rise of multiple insecticide resistance in *An. funestus* s.s. in southern Malawi. This is of great concern for malaria control in this area where *An. funestus* s.s. is the predominant malaria vector. The increase in the resistance level is particularly high in the case of the main insecticides used in malaria control, such as pyrethroids and carbamates. Increased resistance intensity has also recently been reported in *An. gambiae* in Burkina Faso, where in 3 years the resistance ratio increased 1000-fold [37]. Such increase in resistance levels is a concern for the continued effectiveness of insecticide-based control interventions if suitable resistance management is not implemented. Equally, the rise of multiple insecticide resistance in the *An. funestus* population from Chikwawa is of concern as it limits the number of insecticide classes available for IRS. Indeed, the possible resistance to DDT observed in 2009 has now been confirmed in 2014, with only 69.9 % mortality. Resistance to organochlorines also now extends to dieldrin. The confirmation of DDT resistance is a sign of the evolution of resistance patterns in southern African populations of *An. funestus*. The only remaining insecticide class to which no evidence of resistance is seen in *An. funestus* is the organophosphates, which should be recommended for IRS using either malathion or pirimiphos-methyl (Actellic) [38].

### Pyrethroid resistance reduces the efficacy of insecticide-treated bed nets

A worrying observation from this study is the loss of efficacy of bed nets against pyrethroid-resistant *An. funestus* populations from southern Malawi. This loss of efficacy is marked for the net treated only with permethrin (Olyset^®^) while a recovery of efficacy is observed with the net with added PBO (Olyset^®^ Plus). Nevertheless, this synergist net still does not kill 35 % of mosquitoes. This loss of efficacy is similar to that observed in *An. gambiae* in Burkina Faso [37] and suggests that the effectiveness of LLINs might be compromised in areas of high pyrethroid resistance, particularly for LLINs without PBO to block the activity of resistance-associated cytochrome P450s. The results of this study recommend that in such areas of high pyrethroid resistance only nets with PBO be used.

### Change in insecticide resistance mechanisms explains the evolution of resistance

The increased resistance to pyrethroids in this population was associated with increased expression of key cytochrome P450s previously shown to drive resistance, such as *CYP6P9a*, *CYP6P9b* and *CYP6M7* [7, 23]. This further supports previous observations that these genes are the main drivers of pyrethroid resistance in southern African *An. funestus*.

The rise of multiple insecticide resistance in Chikwawa was further supported by the first reported detection of the *RDL* mutation in a southern African population of *An. funestus* in association with dieldrin resistance. This mutation, highly prevalent in West Africa, was not reported in Malawi in 2009 or in any other southern African country [39]. However, the frequency of the 296S-resistant allele is still relatively low (10.52 %), suggesting recent introduction of the allele either as a result of gene flow from populations of other African origin or as a de novo mutation.

The absence of the L119F-GSTe2 DDT resistance mutation [24] in Chikwawa suggests that DDT resistance in southern Africa is driven by a different mechanism to that observed in West and Central Africa. The nearly full recovery of DDT susceptibility observed after PBO exposure suggests that cytochrome P450s are playing an important role, as observed in *An. gambiae* [40] or in *Drosophila* [41].

## Conclusions

The increased resistance levels and rise of multiple resistance reported in here represents a serious challenge to current and future insecticide-based vector control interventions as they limit the choice of alternative insecticides for future interventions. This highlights the urgent need to design and implement suitable resistance management strategies to ensure a continued effectiveness of existing insecticides.

## Additional files

**Additional file 1: Table S1.** List of primers used for Taqman A296S assay and VGSC sequencing.
**Additional file 2: Figure S1.** Gel picture showing the species identification banding patterns obtained by PCR. Lanes are: 1, *An. funestus* s.s.; 2, *An. rivulorum*; 3, *An. rivulorun*-like; 4, Hybrid *An. funestus* s.s./*An. rivulorum*; 5, Hybrid *An. funestus* s.s./*An. rivulorum*-like; 6, Hybrid *An. rivulorum*/*An. rivulorum*-like; 7, *Anopheles parensis*.
**Additional file 3: Table S2.** Summary statistics for polymorphism at the sodium channel gene in susceptible and resistant permethrin and DDT *Anopheles funestus* in Chikwawa, Malawi.
**Additional file 4: Figure S2.** Correlation between haplotype distribution of VGSC gene and resistance phenotypes to DDT and permethrin. (A) Maximum likelihood tree of VGSC haplotypes for both DDT and permethrin-resistant and -susceptible An. funestus from Chikwawa; (B) For DDT alone. AL and DE denote mosquitoes alive or dead after insecticide exposure (i.e., resistant or susceptible). Perm denotes Permethrin.
