# Supplementary material for: Rise of multiple insecticide resistance in Anopheles funestus in Malawi: a major concern for malaria vector control
Source: Malar J. 2015 Sep 15;14:344. doi: 10.1186/s12936-015-0877-y (PMC4570681; doi:10.1186/s12936-015-0877-y)
Supplement: Supplementary file 1 — Additional file 1: Table S1. List of primers used for Taqman A296S assay and VGSC sequencing. [file 12936_2015_877_MOESM1_ESM.pdf]

Table S1: List of primers used for Taqman A296S assay and VGSC sequencing

Primers	Sequence	Modification / Expected size (bp)
Forward	CATATCGTGGGTATCATTTTGGCTAAA	
Reverse	CGACATCAGTGTTGTCATTGTCAAG	
Reporter A296	AGGAGCGTATTCTTTTCTA	VIC
Reporter 296S	AGGAGCGTATTTTTTTCTA	FAM
VGSC-funestus Forward	GTTCAATGAAGCCCCTCAAA	
VGSC-funestus Reverse	CCGAAATTTGACAAAAGCAAA	994
VGSC-arabiensis Forward	AAATGTCTCGCCCAAATCAG	
VGSC-arabiensis Reverse	GCACCTGCAAAACAATGTCA	550
