# Supplementary figures and images for: Rise of multiple insecticide resistance in Anopheles funestus in Malawi: a major concern for malaria vector control
Source: Malar J. 2015 Sep 15;14:344. doi: 10.1186/s12936-015-0877-y (PMC4570681; doi:10.1186/s12936-015-0877-y)

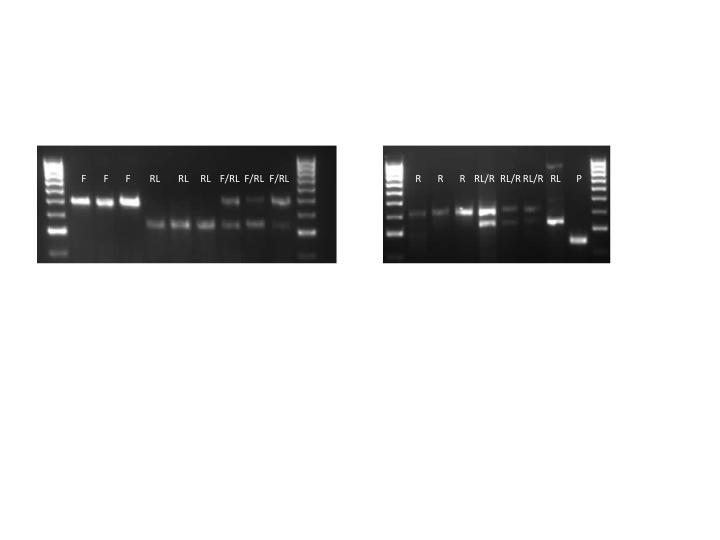

Supplement: Supplementary file 2 — Additional file 2: Figure S1. Gel picture showing the species identification banding patterns obtained by PCR. Lanes are: 1, An. funestus s.s.; 2, An. rivulorum; 3, An. rivulorun-like; 4, Hybrid An. funestus s.s./An. rivulorum; 5, Hybrid An. funestus s.s./An. rivulorum-like; 6, Hybrid An. rivulorum/An. rivulorum-like; 7, Anopheles parensis. [file 12936_2015_877_MOESM2_ESM.tiff]

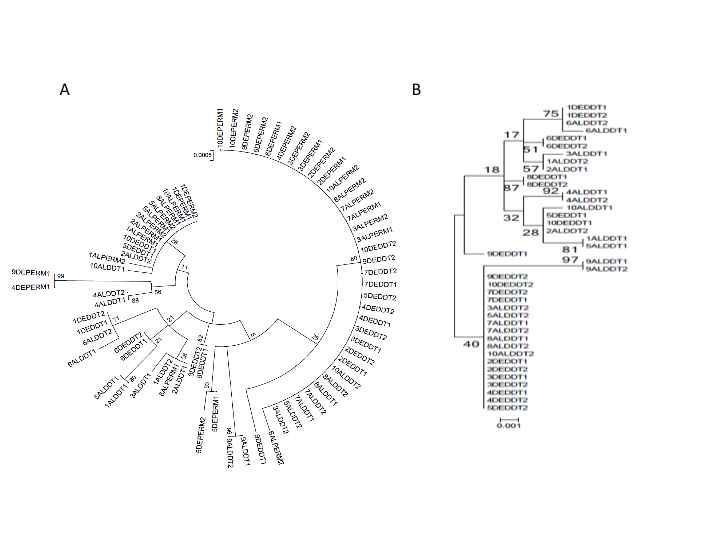

Supplement: Supplementary file 4 — Additional file 4: Figure S2. Correlation between haplotype distribution of VGSC gene and resistance phenotypes to DDT and permethrin. (A) Maximum likelihood tree of VGSC haplotypes for both DDT and permethrin-resistant and -susceptible An. funestus from Chikwawa; (B) For DDT alone. AL and DE denote mosquitoes alive or dead after insecticide exposure (i.e., resistant or susceptible). Perm denotes Permethrin. [file 12936_2015_877_MOESM4_ESM.tiff]
