# Supplementary material for: Rise of multiple insecticide resistance in Anopheles funestus in Malawi: a major concern for malaria vector control
Source: Malar J. 2015 Sep 15;14:344. doi: 10.1186/s12936-015-0877-y (PMC4570681; doi:10.1186/s12936-015-0877-y)
Supplement: Supplementary file 3 — Additional file 3: Table S2. Summary statistics for polymorphism at the sodium channel gene in susceptible and resistant permethrin and DDT Anopheles funestus in Chikwawa, Malawi. [file 12936_2015_877_MOESM3_ESM.pdf]

Table S2: Summary statistics for polymorphism at the sodium channel gene in susceptible and resistant permethrin and DDT *An. funestus* in Chikwawa, Malawi.

Samples	N	S	π (k)	h(hd)	Syn	Nonsyn	D	D*
				**Permethrin**				
**Resistant**	16	9	0.0039(3.65)	5(0.7)	0	0	1.27^{ns}	0.43^{ns}
**Susceptible**	16	14	0.0046(4.3)	5(0.61)	0	0	0.06^{ns}	1.16^{ns}
**Total Permethrin**	32	17	0.0042(3.85)	8(0.68)	0	0	-0.078^{ns}	0.49^{ns}
				**DDT**				
**Resistant**	20	14	0.0053(4.96)	10(0.86)	0	0	0.94^{ns}	1.5*
**Susceptible**	20	10	0.0038(3.54)	6(0.69)	0	0	0.89^{ns}	1.41*
**Total DDT**	40	15	0.0046(4.3)	13(0.79)	0	0	0.7^{ns}	1.5*
**Overall Total**	72	21	0.0045(4.2)	18(0.75)	0	0	-0.05^{ns}	0.95^{ns}

N, number of sequences (2n); S, number of polymorphic sites; h, Number of haplotypes (haplotype diversity); π, nucleotide diversity (k= mean number of nucleotide differences); D and D*, Tajima's and Fu and Li's statistics; ns, not significant; *, P<0.05
